# The more numerous the longer: how the integration between numerosity and time leads to a common neural response

**DOI:** 10.1098/rspb.2023.0260

**Published:** 2023-05-10

**Authors:** Gianfranco Fortunato, Irene Togoli, Domenica Bueti

**Affiliations:** International School for Advanced Studies (SISSA), Via Bonomea 265, 34136 Trieste, Italy

**Keywords:** time, numerosity, perception, ultra-high-field fMRI, population receptive field modelling

## Abstract

If you are stuck in a traffic jam, the more numerous the queuing cars are, the longer you expect to wait. Time and numerosity are stimulus dimensions often associated in the same percept and whose interaction can lead to misjudgements. At brain level it is unclear to which extent time and numerosity recruit same/different neural populations and how their perceptual integration leads to changes in these populations' responses. Here we used high-spatial-resolution functional magnetic resonance imaging with neural model-based analyses to investigate how the topographic representations of numerosity and time change when these dimensions are varied together on the same visual stimulus in a congruent (the more numerous the items, the longer the display time) or incongruent manner. Compared to baseline conditions, where only one dimension was changed at a time, the variation of both stimulus dimensions led to changes in neural population responses that became more sensitive either to the two features or to one of them. Magnitude integration led also to degradation of topographies and shifts in response preferences. These changes were more pronounced in the comparison between parietal and frontal maps. Our results while pointing to partially distinct representations of time and numerosity show a common neural response to magnitude integration.

## 1. Introduction

Every percept unfolds with various extents in a plethora of different dimensions, e.g. space, numerosity and time. Correctly identifying and representing information across different but co-occurring magnitude dimensions is an important function of the brain, and it is essential for effectively interacting with the environment. However, the interaction between different magnitudes can bias our perception and influence our decision: it is always too late when at the supermarket for example, we realize that the cashier queue with the fewest people in line is not the fastest.

Indeed, perceptual biases across magnitude dimensions have been reported in several experimental works [1,2]. In these experiments the estimation of a target magnitude dimension is affected by parametric changes in an irrelevant one. In particular, the interaction between time and numerosity has been shown to influence perceptual judgement asymmetrically [3–5], i.e. numerosity biases duration but duration does not influence numerosity; or symmetrically [6], both magnitudes affecting each other equally. The discrepancies in time and numerosity interactions have been shown to be related to the type of stimuli and tasks employed, suggesting the possibility of different ways in which these two magnitudes can be encoded and integrated [7]. However, the nature of this interaction, thought to be present at the beginning of postnatal life (see for instance [8]), at brain level remains unclear.

An interesting work by Hayashi *et al.* [9] combining functional magnetic resonance imaging (fMRI) with transcranial magnetic stimulation (TMS) experiments characterizes the functional role of the intraparietal cortex (IPC) and inferior frontal gyrus (IFG) in mediating time and numerosity interactions. Using a series of tasks in which the duration and the numerosity of the stimuli were manipulated congruently, the longer the stimulus the higher its numerosity, or incongruently, the longer the stimulus the lower the numerosity, the authors were able to conclude that both regions store a common representation of temporal and numerical information and that the interaction between these two magnitudes occurs at perceptual level in IPC and at a more abstract level in IFG. However, from this but also previous works [4,10–13] it remains unclear whether these interactions originate within neuronal populations tuned to both magnitudes or whether they are caused by the crosstalk of distinct populations of neurons coding for time and numerosity separately. Recent studies, using ultra-high-field fMRI and neural model-based analyses, have shown that the representation of temporal and numerical information is supported, similarly to other low lever stimulus features, by mechanisms of tuning and topography (see [14] for a review). Numerosity and timing maps have been described in a wide network of partially overlapping brain areas, from occipital to parietal to frontal regions [15–17]. However, in these studies time and numerosity were always manipulated separately, leaving unexplored the effect of their interaction on the population tuning.

The present study, by varying parametrically stimulus duration and numerosity, aims to assess if and how the brain responses to time and numerosity change as a function of magnitude integration. The idea, by using neural model-based analyses on high-spatial-resolution fMRI data, is to investigate how the tuning responses to time and numerosity (i.e. the time and numerosity preferences in cortical maps) change as a function of magnitude integration and if these changes affect neuronal populations selective to time, numerosity or both.

## 2. Results

In this study we explored how the tuning properties of brain responses change as a function of the interaction between stimulus magnitude dimensions. Six healthy volunteers were asked to passively appreciate stimuli (dot-arrays) varying in either duration, numerosity (baseline conditions) or both (congruent and incongruent conditions) in different fMRI runs (see Material and methods). In the baseline conditions (time T and numerosity N) we kept one magnitude dimension fixed while varying the other sequentially, in ascending (from lower to higher magnitudes) and descending (from higher to lower magnitudes) cycles (for a visualization of the stimuli space, see electronic supplementary material, figure S2a). The ‘congruent C’ and ‘incongruent I’ conditions were designed to reveal the effect of the interaction of duration and numerosity on brain responses. In the C condition magnitudes varied in the same direction either increasing or decreasing, in the I condition, instead, magnitudes changed in opposite direction: while one increased the other decreased. In all experimental conditions, the magnitude varied sequentially in ascending and descending cycles (see Material and methods).

We studied neuronal population tuning properties by assuming that BOLD responses to our stimuli sequences were generated by a bivariate Gaussian tuning function sensitive to both magnitude dimensions (see Material and methods; and electronic supplementary material, figure S2b). The recorded BOLD signal in each cortical location could be thus described using the five parameters of the population response function: the combination of duration and numerosity in the stimulus space eliciting the greatest BOLD response *μ*_d_, *μ*_n_, the sensitivity of this response *σ*_d_, *σ*_n_ and the orientation of the response function *θ*, which appraises the contribution of each magnitude dimension in generating the tuning response (see electronic supplementary material, figure S2b,c, for model fitting).

We considered only cortical locations whose model fit could explain at least 25% of the variance in the data and belonging to a cluster whose size could appear randomly with less than 1% probability. Electronic supplementary material, figure S3, shows for each cortical location in a common brain space (i.e. Freesurfer's fsaverage) the number of participants in which our modelled response was above this criterion. Given the population level distribution of results we identified six regions of interest (ROIs) on which we focused for further analyses: two occipital, one lateral LO (lateral occipital cortex) and one temporal TO (temporal occipital cortex); two parietal, one in the occipital end of the intraparietal sulcus PO (parieto-occipital cortex) and one in the anterior part of the intraparietal sulcus PC (parietal cortex); and two frontal, one superior FC (frontal cortex) and one inferior IF (inferior frontal cortex). This set of ROIs is very similar to that reported in previous time and numerosity mapping studies [15,17]. The ROI selection on participants' native space was done for each experimental condition independently (see Material and methods).

To understand whether our four experimental manipulations (i.e. T, N, C and I) generated responses in distinct or shared neuronal populations, in each ROI and in each subject, we computed the fraction of vertices on the cortical sheet shared between each pair of experimental conditions. The results of this analysis are reported in electronic supplementary material, figure S4, and showed that duration and numerosity produced distinct and only partially overlapping brain responses (grand average overlap = 0.1205, s.d. = 0.1127). This overlap was higher in occipital (LO = 0.1195, s.e. = 0.0363; TO = 0.1439, s.e. = 0.0367) and parietal regions (PO = 0.1287, s.e. = 0.0364; PC = 0.1206, s.e. = 0.0365) compared to frontal ones (FC = 0.0917, s.e. = 0.0381; IF = 0.0791, s.e. = 0.0367), and it was greater between the baseline conditions (T and N = 0.1337, s.e. = 0.0368) compared to the C and I conditions (C and I = 0.1025, s.e. = 0.0366; the values in this section refer to estimated marginal means and their s.e.; see electronic supplementary material, figure S4 caption, where all the relevant statistics are reported).

**Figure 1.**
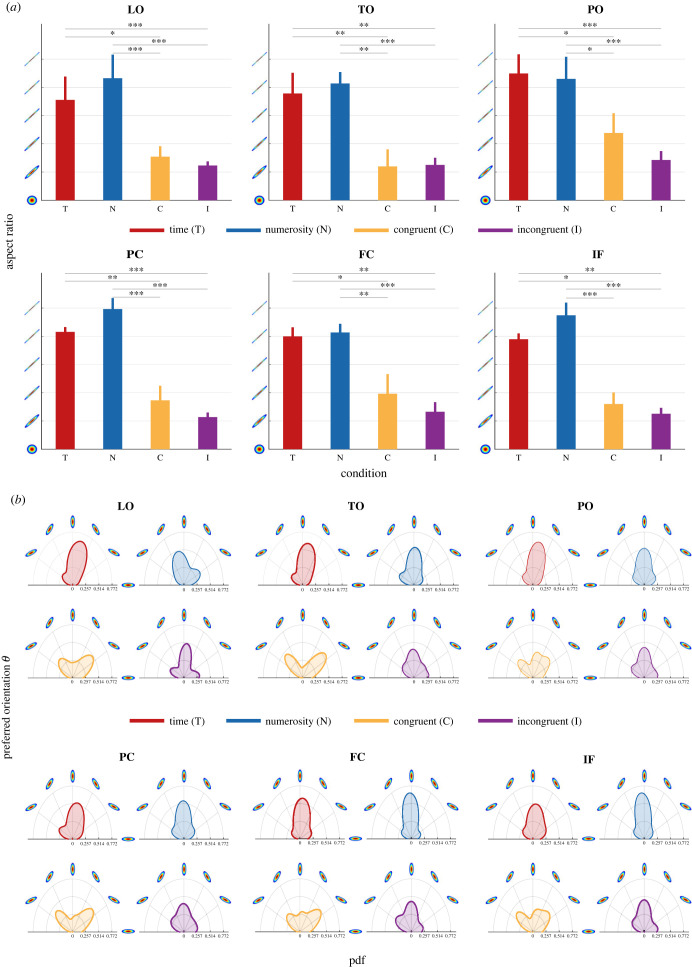
Time and numerosity interaction. (*a*) Bar plots show the mean aspect ratio (*y*-axis) of each map in all conditions (*x*-axis, colour coded as follows: red is T, blue N, yellow C, purple I conditions) averaged across participants. The aspect ratio, computed as the ratio between receptive fields' major and minor axes, characterizes the shape of the receptive field, as graphically depicted in the *y*-axis ticks. Values span from 1, a perfect circle (i.e. equal sensitivity to changes in both dimensions), to 26, a very oblong shape that depending on the orientation may indicate poor sensitivity to changes to either or both dimensions, in steps of 5. The error bars show the standard error of the mean. (*b*) Polar plots show the distribution of the receptive field orientation between 0° and 180° in all the maps of all participants in the different conditions (colour coded as in *a*). The orientation determines whether a vertex responds to variation of either duration or numerosity (when the orientation values are 0°, 90° or 180°) or both (values in between). Distribution kernels were estimated with 15° bandwidth.

We then explored the effect of time–numerosity interaction on brain responses by looking at the distribution of population response parameters within and across ROIs in the different experimental conditions. We first focused on two aspects of the bivariate Gaussian function used to fit the data (see electronic supplementary material, figure S2b): its shape and orientation. Concerning the shape of the tuning function, we computed the aspect ratio of the population response function as the ratio between its major and minor axis (i.e. the ratio between the two *σ* parameters). High values of aspect ratio are associated with an elongated shape of the tuning function indicating a high sensitivity to only one of the two stimulus dimensions. Lower values instead signify a receptive field sensitive to both magnitudes. For each participant and each experimental condition, we averaged the aspect ratios within each ROI (figure 1*a*) and we used these data in a linear mixed effect (LME) model with condition and ROI as factors and subjects as random intercept (see Material and methods). Type III ANOVA with Satterthwaite's method for degrees of freedom on the model results (marginal *R*^2^: 0.574; conditional *R*^2^: 0.587) revealed a main effect of condition (*F*_3,109.12_ = 60.606, *p* < 0.001), no effect of ROI (*F*_5,109.21_ = 0.6831) and no interaction between condition and ROI (*F*_15,109.15_ = 0.2965). In all ROIs the aspect ratio was significantly smaller for the C and I conditions compared to the baseline (i.e. T and N; all *t* < −2.69, *p* < 0.05). This result showed how the interaction between time and numerosity affected the sensitivity of the population response. In the baseline conditions (i.e. T and N) the extremely high value of the aspect ratio (red and blue bars in figure 1*a*) indicates an average population response function that was mainly sensitive to variation of a single stimulus dimension. On the other hand, when time and numerosity were manipulated together (yellow and purple bars in figure 1*a*) the shape of the population response became more rounded and thus more sensitive to changes of both dimensions.

We then looked at the orientation of the receptive field, i.e. the *θ* parameter. The *θ* parameter indicates whether the population response function is oriented toward either one of the two magnitude dimensions. When the *θ* value is around 0°, 90° or 180° it means that the neural response is mainly sensitive to changes of one of the two stimulus dimensions. When instead its value is between 0° and 90°, and 90° and 180°, it means that the response function is sensitive to changes of both time and numerosity. Intuitively, this parameter tells whether duration and numerosity contribute independently or jointly in generating the brain response (i.e. *θ* is directly related to the covariance of the bivariate Gaussian function used). Figure 1*b* shows for all the participants, the distribution of the *θ* parameter in all conditions and ROIs. In the baseline conditions, the response orientations are markedly unimodal pointing towards 90°. When the duration and the numerosity of the stimuli were manipulated together instead, the distributions of response orientation became more composite. In the C condition, they showed two main modes pointing toward 45° and 135°, whereas in the I condition the response orientations were more homogeneously distributed and multimodal (showing three modes). These results show that in the baseline conditions, where duration and numerosity were independent, only one of the two magnitude dimensions (either T or N) contributes to the brain response. Whereas in the I and C conditions where time and numerosity were manipulated together, the pattern of result depended on the association between these features. In the C condition the positive association between duration and numerosity was reflected in a prevalence of orientations tuning sensitive to changes of both magnitude dimensions. In the I condition instead, the negative association resulted in a wider variety of brain response function orientations tuned to either time, numerosity or both. In summary, the results of shape and orientation of the population's tuning function used to model the data clearly show that the simultaneous manipulation of time and numerosity leads to changes in the same neural populations' response profile. This response profile becomes sensitive to changes of the two magnitude dimensions (low values of aspect ratio) and likely reflects the contribution of neuronal populations tuned to both time and numerosity (wider range of *θ*).

**Figure 2.**
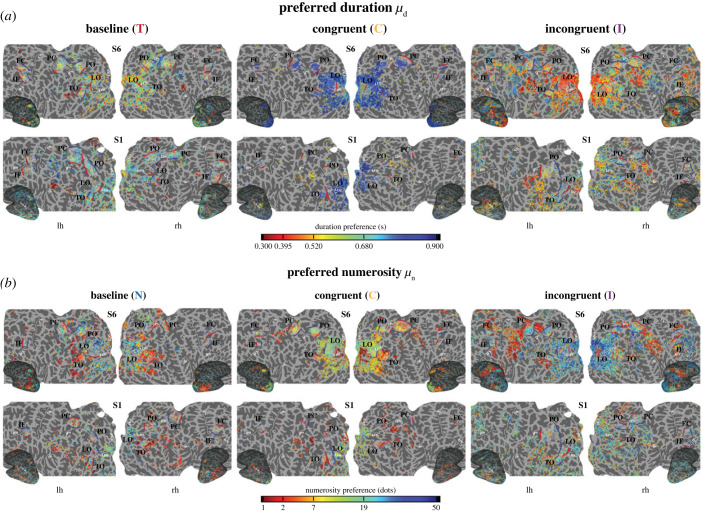
Population receptive field maps of preferred duration and numerosity. Distribution of preferred duration and numerosity (colour-coded) in the different experimental conditions projected onto a flattened and inflated cortical surface of two example participants (each vertex: *R*^2^ > 25%, *p* < 0.01 cluster-level corrected). (*a*) The duration preferences; (*b*) the numerosity preferences. In different columns of the panels are the different experimental conditions (T, N, C and I). Red and blue lines refer to short/low and long/high map edges respectively. Map lateral edges are indicated by white dashed lines. Solid white lines mark principal sulci. Legend: LO = lateral occipital, TO = temporal occipital, PO = parieto-occipital, PC = parietal cortex, FC = frontal cortex, IF = inferior frontal cortex, LOS = lateral occipital sulcus, IPS = intraparietal sulcus, CS = central sulcus, SF = Sylvian fissure, IFS = inferior frontal sulcus, lh = left hemisphere, rh = right hemisphere.

We next focused on the distribution of response preferences in the different conditions. Figure 2 shows the distribution of preferred duration *μ*_d_ (figure 2*a*) and preferred numerosity *μ*_n_ (figure 2*b*) on the flattened and inflated representation of the cortical surface in two example participants. For both baseline conditions, we found in the selected ROIs a distribution of preference topographically organized across the cortical surface. Interestingly time and numerosity maps changed considerably when the two magnitudes were manipulated together. We analysed these changes within and across ROIs as well as their topographical organization using LME models (see Material and methods).

Within each ROI and for both duration and numerosity preference the employed LME models were able to explain the data well (median marginal *R*^2^: 0.5442, median conditional *R*^2^: 0.6462) and they showed that duration and numerosity preference changed in the different experimental conditions (main effect of condition all *F* > 10.9, *p* < 0.001). Duration and numerosity preference changed depending on the position of the vertices in the maps (main effect of distance all *F* > 68.9, *p* < 0.001) and these changes were different in the different conditions (interaction between condition and distance all *F* > 5.8, *p* < 0.001).

**Figure 3.**
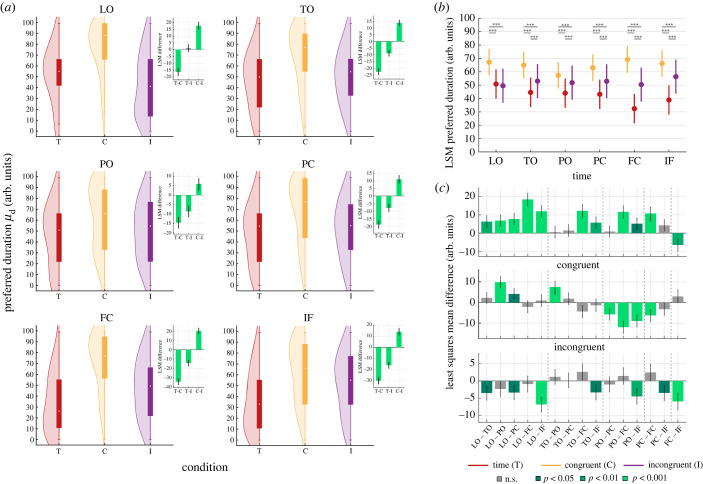
Distribution of preferred duration (*μ*_d_) in the different maps. (*a*) Violin plots show the distribution of preferred duration (*μ*_d_) of all participants in the different experimental conditions colour-coded as in figure 1 (arb. units is arbitrary units). Solid bars represent the interquartile ranges. The extremes of the whiskers are the minimum and maximum values of the distributions, the white circles refer to the distributions' medians, distributions’ kernels were estimated with 15 arb. units bandwidth. The insets show the least-squares mean differences between conditions in each map. (*b*) The least-squares mean preferred duration (*y*-axis) across all maps (*x*-axis) in the different experimental conditions (colour coded). (*c*) The least-squares mean differences in preferred duration shown as difference between ROI pairs. Bars are colour-coded according to the *t* statistics significance level. All comparisons are Bonferroni-corrected for multiple comparisons. Error bars represent 95% bootstrapped confidence intervals. ROI legend as in figure 2.

**Figure 4.**
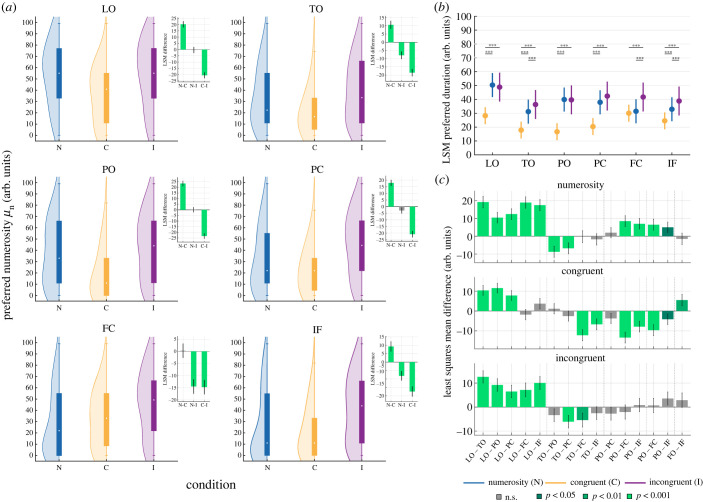
Distribution of preferred numerosity (*μ*_n_) in the different maps. The same as figure 3 for numerosity preference.

As shown by figure 3*a* when the duration and the numerosity of the stimuli were manipulated together, population responses were higher for the longer durations as compared to the baseline condition. This shift in the average duration preference was present in the C condition, where the more numerous the stimulus the longer its duration (T–C all *t* < −9.4, *p* < 0.001) and to a lesser extent in the I condition (T–I all *t* < −5.7, *p* < 0.001). This shift concerned all ROIs except LO where in the I condition we found no shift (see the insets in figure 3*a*). On the contrary (figure 4*a*), the average population response preference shifted towards smaller numerosity when stimulus duration and numerosity varied congruently (N–C all *t* > 6.1, *p* < 0.001, a non-significant shift in preference was found only in the FC ROI). In the I condition, where longer display times corresponded to fewer dots in the stimulus, numerosity preferences shifted towards higher numerosity in TO, FC and IF ROIs (N–I all *t* < −5.81, *p* < 0.001).

In addition, we checked how duration and numerosity preferences changed across ROIs (figures 3*b* and 4*b* respectively). Overall, in both the baseline conditions (T and N), we found a general decrease in numerosity and duration preferences moving from LO to TO; preferences remained stable in PO and PC and they decreased again in FC and IF (figures 3*b*, 4*b* and 3*c*, 4*c*). In the C condition the pattern of preference change was similar to the baseline conditions from LO to PO–PC for both duration and numerosity preference, although this decrease was more prominent for numerosity, and it switched to an opposite trend from PO–PC to IF (figures 3*b*, 4*b* and 3*c*, 4*c*). The distributions of preferences in the I condition remained more stable across ROIs, moderately increasing from the occipital to the frontal ROIs in case of duration preference (figure 3*c*) and decreasing from the occipital to the parietal ROIs, in the case of numerosity preference. In this latter case, no significant change was found from parietal to frontal regions (figure 4*c*). All the statistics of this analysis are reported in the electronic supplementary material.

In summary, these results show that the combination of time and numerosity in the same visual stimulus led to changes in response preferences. These changes were in opposite direction for time and numerosity: while duration preference increased in both C and I condition, numerosity preference decreased in the C and increased in the I condition. These changes also affected response preferences in the different ROIs: while at baseline both numerosity and duration preferences decreased from occipital to frontal regions, this pattern was reversed (i.e. preferences increased) in the C condition. This shift in preference happened in the comparison between parietal (PO, PC) and frontal regions (FC and IF) suggesting the presence of a two-stage mechanism of magnitude processing and integration along the cortical hierarchy.

After having assessed changes in preference distribution, we checked how the interaction between time and numerosity affected the topographical organization of preferred duration and numerosity.

**Figure 5.**
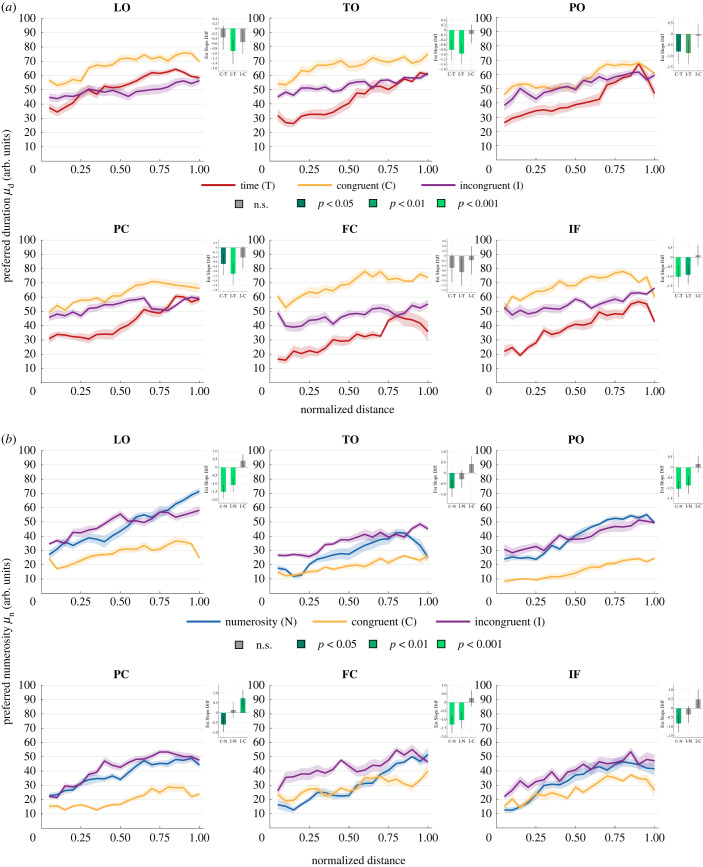
Topographic arrangement of duration and numerosity maps in different conditions. The curves in each subplot are the mean across participant of (*a*) preferred duration (*μ*_d_) and (*b*) preferred numerosity (*μ*_n_) as function of normalized cortical distance (see Material and methods). Each subplot is a different ROI and colour-coded are the different experimental conditions (arb. units is arbitrary units). The shaded areas represent the standard error of the mean. The insets display the difference in the estimated progression slopes. The bars are colour-coded according to *t* statistics significance level Bonferroni-corrected for multiple comparisons (from dark green *p* < 0.05 to light green *p* < 0.001, grey is not significant). Error bars represent 95% bootstrapped confidence intervals. ROI legend as in figure 2.

Figure 5 shows in the different ROIs how the spatial progression (i.e. the distance of a vertex from the map's border) of duration (figure 5*a*) and numerosity (figure 5*b*) preferences change in the different experimental conditions.

To be able to quantify the topographical organization of the preferred duration and numerosity, in each individual subject and for each ROI and condition we draw on the cortical surface a set of map borders. For each vertex of the cortical surface (within each ROI) we computed its normalized distance (i.e. the ratio between the vertex distance from one edge of the map and the distance between the two map edges; see Material and methods) from one of these borders, i.e. the low/short border. We then grouped vertices based on their normalized distance (from 0 to 1 in steps of 0.05) and averaged their preferred duration or numerosity (figure 5).

We used the slope of these progression curves as an index of the quality of the maps: the steeper the slope the better the map (i.e. the clearer the change in preference and the wider the range covered within the boundary of the ROI). Statistical assessment of the differences in progression slopes between conditions in the different ROIs was done using the same LME model described in the previous paragraph (see also Material and methods). For duration preference, the insets in each plot show that the progression slopes were significantly flatter compared to baseline when time and numerosity were manipulated together. The only exceptions were LO and FC ROIs (all the other *t* < −2.981, *p* < 0.02). The topographic organization of numerosity preference instead remained similar to the baseline in the I condition, where only three out of six ROIs showed a flatter progression slope (all *t* < −4.116, *p* < 0.001), and it became consistently degraded in the C condition (all *t* < −2.95, *p* < 0.02). These results show that the interaction between time and numerosity affected not only the overall distribution of preferences of the population response but also their topographic organization. These changes though affected time and numerosity maps to a different degree. The spatial progression of time maps degraded in both C and I conditions, whereas that of numerosity maps degraded in the C condition only.

Finally, to make sure that our results were not just the byproduct of our fitting procedure, we tried to retrieve a signature of Weber's law (i.e. the higher the preference, the lower the sensitivity—the bigger the *μ* the bigger the *σ*) from the distribution of the response function parameters in the different experimental conditions. Given our experimental manipulation and given the function with which we described the population tuning, we reasoned that the dependence between the preferential response (*μ*) and the sensitivity (*σ*) would be dependent on the orientation of the response function (*θ*). For this reason, in each ROI and for each condition, we divided the vertices in 5 groups based on their preferred orientation. Three of these orientations, i.e. *θ* = 0°, 90° and 180°, refer to a tuning function mainly sensitive to changes of a single stimulus dimension, the other two, i.e. 45° and 135°, reflect a tuning function sensitive to both time and numerosity (see Material and methods). For each pair of *μ* and *σ* we computed Kendall's tau correlation coefficient. The results of these correlations for the different experimental conditions are shown in electronic supplementary material, figures S7 (for the two baselines) and S8 (for C and I conditions). As expected, in the baseline conditions for 0°, 90° and 180° orientations which are highly represented in these conditions (see the *θ* distribution of figure 1*b*), the scalar property was evident, a single *σ* parameter was positively correlated with a single *μ* parameter (*σ*_d_ with *μ*_d_ in T condition and *σ*_n_ with *μ*_n_ in N condition when *θ* = 0°, whereas *σ*_d_ with *μ*_n_ in time condition and *σ*_n_ with *μ*_d_ in numerosity condition when *θ* = 180°—see electronic supplementary material, figure S7). For the C and I conditions, depending on the sensitivity of the tuning functions (i.e. the *θ* distribution in figure 1*b*) which comprises a variety of orientations, we would expect different results. For 0°, 90° and 180° orientations we would imagine the appropriate *σ* to correlate with the appropriate *μ*, compatibly with the idea that there are distinct neuronal populations tuned to either time or numerosity and that they are both active in C and I conditions. Electronic supplementary material, figure S8, shows this kind of pattern of correlations for the I condition only. On the contrary, in the C condition, we observed strong correlations of *σ*_d_ and *σ*_n_ with a single *μ* parameter, suggesting the presence of a population response mostly informed by a single magnitude dimension. For orientations close to 45° and 135°, thus for tuning functions sensitive to changes of both dimensions, if both stimulus dimensions contribute equally to the population response, their combination should be a single dimension and thus a single *σ* parameter should positively correlate with both duration and numerosity preferences (*μ*_d_ and *μ*_n_). We found the expected pattern in the I condition only. In the C condition instead, we found the opposite pattern, i.e. both *σ*_d_ and *σ*_n_ correlating with a single *μ*, suggesting that the population tuning was mainly driven by a single stimulus dimension. These latest results show that the response function we used to model the data was able to capture the presence of the scalar property and together with the results of orientation and shape of the tuning function (figure 1) seem to suggest the idea that in conditions where time and numerosity are manipulated together, the brain response is driven by units selective to either one or two stimulus dimensions.

## 3. Discussion

In this work we studied the effect of time and numerosity interaction on the response properties of six regions of interest spanning from occipital to frontal cortex. In the baseline conditions, where time and numerosity were manipulated independently, we found duration and numerosity maps in cortical locations very similar to those identified by previous studies [15,17]. Moreover, similarly to Harvey *et al*. [17], who did not use an explicit temporal task, we did not observe duration maps in SMA [16]. The lack of a temporal task might be the cause of this absence.

In agreement with these *previous* studies, we also found that duration and numerosity elicited partially overlapping (particularly in occipital and parietal regions) but distinct brain responses, supporting the idea that duration and numerosity maps subserve quantity-specific mechanism of information representation. Previous fMRI experiments have shown indeed that visual and haptic maps of numerosity [15,18], visual maps of size and time [17,19] although they partially overlap within the parietal cortex, they differ in their topographic organization (see [14]). Our work not only confirms those previous studies by showing a partially independent representation of time and numerosity (i.e. only partially overlapping maps), but goes beyond them showing for the first time, that when these magnitudes are manipulated together in the same visual stimulus they elicit brain responses that reflect neural populations sensitive to either one or both these features. The shape of the tuning functions becomes indeed rounder (i.e. lower aspect ratio) while its orientation broadens. In this respect there seems to be a difference between C and I conditions. In the C condition where time and numerosity are positively correlated, the orientation of the tuning functions seems to suggest the presence of a brain response sensitive to variations of both features. However, when we looked at the relationship between response preference (*μ*) and variance of the tuning curves (*σ*), we observed in most of the ROIs that the sigma parameter for time and numerosity (*σ*_d_ and *σ*_n_) correlated with a single *μ* suggesting that the population tuning was mainly driven by a single stimulus dimension. As if, due to the positive correlation between time and numerosity, a single dimension drives the response. On the other hand, in the I condition, where the two dimensions are anticorrelated, the orientations of the tuning curves are mixed. There are orientations suggesting response preference tuned to either time or numerosity and where we observe the expected correlation between mean preference and variance (e.g. *σ*_d_–*μ*_d_ and *σ*_n_–*μ*_n_ at *θ* = 0°). While other orientations, suggesting response selectivity to both time and numerosity, have a single *σ* parameter positively correlating with both duration and numerosity preferences (*μ*_d_ and *μ*_n_). As if time and numerosity contribute equally to the population response. Even if the difference observed between C and I condition could be explained by the way time and numerosity are combined in the stimuli, the absence of a task or of an attentional manipulation prevents us from saying a conclusive word on this matter.

When time and numerosity were manipulated together, even response preferences and the topographical arrangement of the maps underwent drastic changes compared to the baseline conditions. The spatial progression of duration maps became significantly degraded in both C and I conditions while numerosity maps showed a degraded topographical distribution in the C condition only. These topographical changes might reflect the changes in neural population responses that either become sensitive to changes of the two stimulus dimensions or keep the tuning to one of them.

Concerning response preferences, the distribution of duration preferences shifted towards longer durations in both C and I conditions whereas the distribution of numerosity preference shifted toward shorter numerosities in the C condition and longer numerosities in the I one. Despite the absolute difference between numerosity and time response preference shifts in C and I conditions, these shifts had a similar pattern across ROIs for both magnitude dimensions. From the occipital (LO) to the parietal cortex (PO and PC), in fact, both the average duration and numerosity preference decreased in all conditions, suggesting the presence of shared, or at least similar, processing of magnitude information. At this stage, duration and numerosity information might be integrated within a unitary representation of the stimulus in the parietal cortex [20,21]. The differences between conditions (i.e. baseline versus either C or I condition) appeared in the comparison between parietal (PO and PC) and frontal (IF) ROIs. In the C condition both duration and numerosity preferences increased moving from PC to FC and IF. In the I condition instead, this pattern was similar for numerosity but not for duration preference where there was no significant change across ROIs. The fact that the shift in response preference happened between parietal and frontal regions may suggest the existence of a two-stage mechanism of magnitude processing and integration: an early encoding stage involving parietal cortex and a late perceptual and decision-dependent stage in frontal cortex. Empirical support to the hypothesis of a two-stage mechanism of magnitude integration and representation comes from a study by Hayashi and colleagues [9]. By comparing subjects’ performance before and after continuous theta-burst stimulation in different tasks, those authors found that the degree of interaction between time and numerosity was modulated only after the stimulation of the right intraparietal cortex and not of the inferior frontal gyrus. The authors proposed that a common perceptual representation of magnitude information is formed within the parietal cortex and then transformed into a more abstract, categorical representation in the frontal regions. Similarly, our results highlight the role of parietal cortex as the key point of transition between an early encoding stage and a late perceptual and condition-dependent stage of magnitude processing. If confirmed in future studies, this result could potentially explain both the symmetric and the asymmetric perceptual effects of the interaction between time and numerosity [4,7,22].

The reason why the response preferences shift toward a certain direction in C (increasing in duration and decreasing in numerosity) or I conditions (increasing in both duration and numerosity) is difficult to establish. The absence of a perceptual task does not allow the identification of the possible sources of these shifts in preference. The changes in duration and numerosity preferences might reflect a genuine perceptual bias or alternatively a more general effect of feature attention which is known to modulate magnitude integration (see [23,24]). A recent study has indeed shown that attention is required to elicit tuned response to numerosity. By asking participants to pay attention only to a subset of dots (white or black) in a dot array, Cai *et al.* [25] have shown that brain responses are suppressed for these unattended numerosities.

Even considering these limitations, we believe our results clearly support the idea that the brain mechanisms underlying magnitude representation are not independent. Indeed, the pattern of brain response is not only determined by each magnitude separately, but also by the conjunction of them when they change together. Importantly, our results reveal a pivotal role of the context in which a stimulus is presented (i.e. C versus I) in determining whether and to which extent the cortical representation of time and numerosity changes as a function of magnitude integration.

Overall, our results suggest that neuronal populations sensitive to a single magnitude dimension might coexist with populations sensitive to both, and that overall brain response became more sensitive to both numerosity and duration when these features are manipulated together.

## 4. Material and methods

### (a) Participants

Six healthy volunteers took part in this study (mean age: 25.4, s.d.: 5.4, 3 females, one left-handed participant was also included in the sample). All volunteers gave written informed consent to participate in this study, the procedures of which were approved by the International School for Advanced Studies (SISSA) ethics committee (protocol number 1899/II-16) in accordance with the Declaration of Helsinki.

### (b) Stimuli and procedure

Volunteers underwent four experimental runs, each composed of 130 trials. In each trial we presented on a screen (Cambridge Research 32′ LCD BOLD screen, 1920 × 1080 pixels resolution, 69.8 × 39.3 cm active area and 120 Hz refresh rate) placed at the end of the bore and viewed via a mirror (total viewing distance: 151.5 cm), an array of slow moving dots (velocity = 2° per second) characterized by a specific duration (display time) and numerosity (number of dots in the array). In other to exclude the possibility that the density of the array would contribute to the brain response to numerosity, the stimulus presentation area was varied trial by trial, in a random and counterbalanced order, within 7°, 7.5° and 8° from the screen centre. The starting position of each dot was chosen randomly within the presentation area. Dot sizes were also selected randomly trial by trial with the constraint of keeping their sum constant so that dots' total area was constant across numerosities. Dots’ radii could span from a minimum of 0.31° to a maximum of 2.88°. Dots moved uniformly, with constant velocity, throughout the trial, their motion directions being chosen randomly at the beginning of each trial from a uniform distribution spanning from 0 to 2π rad and in the case of collisions a perfectly elastic rebound was applied. All dot arrays had an equal proportion of black and white dots presented on a grey background.

In each run stimuli were presented in cycles of ten trials; in each run we had a total of 13 cycles. In each cycle dot arrays could change sequentially trial by trial in either duration or numerosity or in both duration and numerosity. The inter-trial interval was set to 1.5 s (1.2 TRs) whereas the inter cycle interval was 5 s (4.1 TRs). The beginning of each cycle was synchronized with the scanner acquisition. Volunteers were asked to press a button on the keypad when a target (i.e. a red dot) was presented in the array. The target was presented on 10% of the trials (once per cycle) in a pseudo-randomized and counterbalanced fashion.

The way in which the stimuli varied in each cycle characterized our four experimental conditions. Only one cycle type was presented per run.

In two runs numerosity and time were varied independently, those representing the baseline conditions. In these runs stimuli within each cycle varied in one magnitude only while the other was kept constant. In the duration baseline (T) stimuli had a fixed numerosity of 100 dots and their duration gradually increased from 0.3 to 0.9 s in five trials and then gradually decreased from 0.9 to 0.3 s in the subsequent five trials. The durations tested were 0.3, 0.395, 0.52, 0.68 and 0.9 s. In the numerosity baseline (N) stimuli had a fixed duration of 0.2 s while their numerosity increased from 1 to 50 dots in the first five trials and from 50 to 1 in the subsequent ones. The numerosities tested were 1, 2, 7, 19, 50. The other two runs were designed to reveal the effects of the interactions between duration and numerosity on population tuning. In the congruent (C) conditions the increase and decrease in numerosity and duration within each cycle were paired so that both increased or decreased. In the incongruent (I) condition instead, the changes in numerosity and duration had opposite sign: while one magnitude was increasing the other was decreasing and vice versa. In the first five trials of a cycle stimulus duration was decreasing from 0.9 to 0.3 s while numerosity was increasing from 1 to 50 dots and in the last five trials of the cycle was the reverse, i.e. the duration was increasing from 0.3 to 0.9 s and the numerosity decreasing from 50 to 1 dot. The presentation order of each condition was pseudo-randomized between subjects. Subjects were unaware of the experimental conditions.

### (c) Magnetic resonance imaging acquisition

We acquired MRI data on a head-only 7 T MRI scanner (Siemens, Germany), equipped with a head gradient-insert (AC84, 80 mT m^−1^ maximum gradient strength; 350 mT m^−1^ slew rate) and a 32-channels receive coil with tight transmit sleeve (Nova Medical, Massachusetts, USA). T2*-weighted functional images were acquired using SMS acquisition with voxel resolution of 1.5 mm isotropic, with a matrix size of 146 × 146 × 75, which resulted in a field of view of 219(AP) × 219(LR) × 112.5(FH) mm. Repetition time (TR) was 1.25 s, echo time (TE) was 0.023 s, flip angle was 60° and bandwidth was 1903 Hz Px^−1^. Slices were oriented transversally with an anterior-to-posterior phase-encoding direction. Numerosity runs contained 265 TRs whereas the other runs 304. Additionally, at the end of each run we acquired 3 volumes with the opposite phase encoding direction. High-resolution T1-weighted images were also obtained using MP2RAGE pulse sequence optimized for 7 T (voxel size = 0.6 × 0.6 × 0.6 mm, matrix size = 320 × 320 × 256, TI_1_/TI_2_ = 750/2350 ms, *α*_1_/*α*_2_ = 4/5°, TR_MP2RAGE_/TR/TE = 5500/6000/4.94 ms).

### (d) Functional magnetic resonance imaging preprocessing

We preprocessed the data using *fMRIPrep* 20.1.1 [26] (see electronic supplementary material, methods). In addition, we high-pass filtered the BOLD time-series by removing the first 6 components from the discrete cosine transform of the data, and we computed the percentage signal change of the resulting filtered time-series.

### (e) Population receptive field modelling

The tuning properties of the brain response were estimated via population receptive field (pRF) modelling [27]. In order to capture fMRI signal change related to stimulus change in both duration and numerosity as well as during their interaction, we used a bivariate Gaussian function as model of neuronal response (i.e. its receptive field):

$$nr\;∼\;N(\mu_{d},\mu_{n},\sigma_{d},\sigma_{n},\theta ),$$

where *μ*_d_ and *μ*_n_ represent, respectively, the preferred duration and numerosity of the receptive field (i.e. the stimulus duration and numerosity eliciting the largest neuronal response), *σ*_d_ and *σ*_n_ the standard deviations along its axis and *θ* its orientation. Fitting procedures were done using custom-made functions of prfpy package [28] (see electronic supplementary material, methods).

During the fitting procedures, stimulus duration and numerosity were represented in arbitrary units (from 0 to 100) to ease comparisons between duration and numerosity maps.

In the two baseline conditions where only one magnitude was manipulated at a time, we made the neuronal response function invariant to the irrelevant magnitude dimension, so that the performance of the receptive field model was not dependent on the fit of the irrelevant *μ* parameter.

For further analyses we kept only the winning models that could explain at least 25% of variance of the measured fMRI time-courses and with model parameters within the stimulus range. This threshold was set to be in line with previous reports of timing and numerosity mapping [17]. In addition, we performed a cluster permutation procedure based on the variance explained by those winning models. We removed from the results clusters of vertices whose sizes had more than 1% probability of appearing by chance. We randomized the location of the above threshold models 1000 times in each condition, hemisphere and subject separately. We then computed the probability distribution associated with the maximum cluster sizes at each iteration using this information to remove from the analysis spurious results.

### (f) Regions of interest selection

We limited the analysis of pRF model parameters to 6 ROIs. Those ROIs were chosen by applying the fitting procedures described in the previous paragraph, on the subjects' fMRI signal resampled on Freesurfer's fsaverage surface. The number of subjects showing the presence of above-threshold fit in each experimental condition (T, N, C, I) was then rendered on a common surface (see electronic supplementary material, figure S3). Guided by the group level results, we selected two occipital ROIs (i.e. LO and TO), two parietal ROIs (i.e. PO and PC) and two frontal ROIs (i.e. FC and IF). Those regions were also consistent with previous reports of time and numerosity maps [15,17].

We used two criteria for ROI identification in the subjects' native space: *map continuity* (i.e. each map should have vertices belonging to the same cluster) and *progression continuity* (i.e. map preference should show only one directionality of the preference gradient). In cases where the results were sparse, we favour the latter criterion to the former. Individual ROI definition was also atlas guided (i.e. each map should belong to a specific atlas-based region characterization). Specifically, we applied on subjects’ cortical surface Wang [29] and Benson [30] atlases using *neuropythy* package [31] which both provide a characterization of visual and parietal regions. In addition, we made use of the Destrieux atlas [32]. Electronic supplementary material, table S1, shows the percentage of vertices within each defined ROI belonging to regions described in the above-mentioned atlases. For each participant we selected our six ROIs in both hemispheres. In total 249 out of 288 (86.5%) possible ROIs were delineated, none of the participants showed the full set of 48 ROIs in both hemispheres in all conditions (41.5 out of 48 on average, spanning from 38/48 in the left-handed participant to 47/48), participants showed on average 5.1 maps out of 6 per hemisphere across conditions. Starting with the baseline conditions, for each ROI we drew four delimiting edges: two lateral edges and two edges marking the low and high end of the preferred magnitude. We labelled those edges as short and long in the case of duration preference and as low and high in the case of numerosity preference. In the C and I conditions since duration and numerosity preference could potentially change differently along the cortical surface, we first marked the lateral edges of the ROI by carefully applying the progression continuity criterion for both duration and numerosity preference simultaneously. We then marked the low/short and the high/long edge of the ROI arbitrarily. This was done for the sole purpose of quantifying vertices' distances within the ROI (see Distance quantification). Before running our statistical analysis we reassigned the low/short and high/long borders based on a data driven approach (see Analysis of duration and numerosity preference within ROI). Electronic supplementary material, figure S4, shows the overlap between conditions in the different ROIs.

### (g) Distance quantification

In order to study changes in parameter distribution within each ROI we computed, for each vertex, its relative distance from the low/short edge. We first flattened the cortical surface of each ROI using Freesurfer's *mris_flatten*, then we found the projections of each vertex on the low/short (A) and high/long (B) edge of the ROI. We then computed the vertex projection (V) on the secant segment between the two edge projections ($\bar{\mathrm{AB}}$). The ratio between the distance from the low/short edge to the vertex's secant projection ($\bar{\mathrm{AV}}$) and the length of the secant segment gave us the vertex's normalized distance from the low/short edge of the ROI ($\mathrm{normalized}\;\mathrm{distance}=\bar{\mathrm{AV}}/\bar{\mathrm{AB}}$).

We then assigned a label to each vertex based on their normalized distance value: vertices were assigned to the same label if they fell with a range of 0.05 normalized distance.

### (h) Analysis of duration and numerosity preference within ROI

In each ROI we compared the model preference to duration and numerosity (*μ*_d_ and *μ*_n_) between the different experimental conditions (T, N, C, I) with LME models, using the *lme4* R package [33]. We built a dataset in which each entry was vertices' mean preference (in duration or numerosity) per distance bin in each subject and condition. Before running the analysis we checked in the C and I ROIs, using linear regression, whether both duration and numerosity preferences were increasing as a function of distance (model formula *μ* ∼ Distance). In the case in which preferences were decreasing as a function of distance we reversed the order of the preference progression. This was done to remove possible inflations in the interaction term of the LME due to mislabelling of ROI edges in the C and I conditions.

The LME model formula was

μ∼Condition∗Distance+(1|subjectID).

The preference was thus explained in terms of the interaction between condition and relative distance from the low/short edge of the map. We included subjects as random intercept in the model. We used Satterthwaite's method [34] for estimating degrees of freedom for model ANOVA using the *lmerTest* package [35]. We computed the difference in the estimated marginal means (or least-squares means) as well as its 95% confidence interval using the *difflsmeans* function (see insets in figures 3*a* and 4*a*). In this case degrees of freedom were estimated using the Kenward–Roger method [36]. The difference in progression slopes (see insets in figure 5*a*,*b*) represents the difference in the model interaction terms between conditions. The 95% confidence intervals in this case were computed with 999 iterations bootstrapping using *confint* R function. All reported *p*-values were Bonferroni corrected for multiple comparisons. LME model variance explained was computed using *MuMIn* package [37].

### (i) Analysis of duration and numerosity preference across ROI

A similar pipeline was employed to study the difference in preference across ROIs. In this case we used ROI as explanatory variable in the LME model for each condition separately:

μ∼ROI∗Distance+(1|subjectID).

The least-squares means were computed per ROI (see figures 3*b* and 4*b*) as well as the least-squares mean differences (see figures 3*c* and 4*c*) as described in the previous paragraph. All reported *p*-values were Bonferroni corrected for multiple comparisons.

### (j) Aspect ratio analysis

We used an LME model to analyse the differences in the shape of the receptive field across conditions and ROIs. In each subject and ROI, we computed the mean response function aspect ratio (i.e. the ratio between its major and minor axis) for each experimental condition. This dataset was used for the LME:

AspectRatio∼ROI∗Condition+(1|subjectID),

to model the effect of ROI and condition as well as their interaction on the shape of the receptive fields. The difference in estimated means is reported in figure 1*a* following the methodology described in the previous paragraphs. All reported *p*-values were Bonferroni corrected for multiple comparisons.

### (k) Correlation between sensitivity and preference

We performed a correlation analysis between the receptive field preferences (*μ*_d_ and *μ*_n_) and sensitivity (*σ*_d_ and *σ*_n_) to investigate whether the latter scales with preferred duration or numerosity according to Weber's law. To this aim, in each subject and ROI, we grouped vertices based on their receptive field orientation (from 0° to 30°, from 30° to 60°, from 60° to 120°, from 120° to 150°, from 150° to 180°). For each group of receptive fields in each ROI we computed the Kendall's tau correlation coefficient between the *μ* and *σ* parameters. These groups were chosen because they represent receptive fields responding to changes in either one (when oriented around 0°, 90° and 180°) or both (when oriented around 45° or 135°) magnitudes. The results of this analysis are shown in electronic supplementary meterial, figures S7 and S8.

## Data Availability

Data and analysis scripts described in this paper are available on Open Science Framework at https://osf.io/ud5sm/ [38]. The data are provided in electronic supplementary material [39].
